# Altered hippocampus and amygdala subregion connectome hierarchy in major depressive disorder

**DOI:** 10.1038/s41398-022-01976-0

**Published:** 2022-05-19

**Authors:** Yael Jacob, Laurel S. Morris, Gaurav Verma, Sarah B. Rutter, Priti Balchandani, James W. Murrough

**Affiliations:** 1 Depression and Anxiety Center for Discovery and Treatment, Department of Psychiatry, Icahn School of Medicine at Mount Sinai, New York, NY USA; 2 BioMedical Engineering and Imaging Institute, Icahn School of Medicine at Mount Sinai, New York, NY USA; 3 Department of Psychiatry, Icahn School of Medicine at Mount Sinai, New York, NY USA; 4 Department of Diagnostic, Molecular and Interventional Radiology, Icahn School of Medicine at Mount Sinai, New York, NY USA; 5 Department of Neuroscience, Icahn School of Medicine at Mount Sinai, New York, NY USA

**Keywords:** Neuroscience, Depression

## Abstract

The hippocampus and amygdala limbic structures are critical to the etiology of major depressive disorder (MDD). However, there are no high-resolution characterizations of the role of their subregions in the whole brain network (connectome). Connectomic examination of these subregions can uncover disorder-related patterns that are otherwise missed when treated as single structures. 38 MDD patients and 40 healthy controls (HC) underwent anatomical and diffusion imaging using 7-Tesla MRI. Whole-brain segmentation was performed along with hippocampus and amygdala subregion segmentation, each representing a node in the connectome. Graph theory analysis was applied to examine the importance of the limbic subregions within the brain network using centrality features measured by *node strength* (sum of weights of the node’s connections), *Betweenness* (number of shortest paths that traverse the node), and *clustering coefficient* (how connected the node’s neighbors are to one another and forming a cluster). Compared to HC, MDD patients showed decreased node strength of the right hippocampus cornu ammonis (CA) 3/4, indicating decreased connectivity to the rest of the brain, and decreased clustering coefficient of the right dentate gyrus, implying it is less embedded in a cluster. Additionally, within the MDD group, the greater the embedding of the right amygdala central nucleus (CeA) in a cluster, the greater the severity of depressive symptoms. The altered role of these limbic subregions in the whole-brain connectome is related to diagnosis and depression severity, contributing to our understanding of the limbic system involvement in MDD and may elucidate the underlying mechanisms of depression.

## Introduction

Major depressive disorder (MDD) is one of the most prevalent health problems, it affects almost 7% of the United States adults and is associated with significant day-to-day life issues, disability, and heavy social and economic burden [1]. It is a complex disorder with heterogeneous clinical manifestations involving affective, cognitive, and somatic symptoms. Currently, the underlying brain pathology remains largely unclear. Accumulating evidence from the last decade suggests that the complex etiology of psychiatric disorders such as MDD are not localized to a single brain region’s morphology, but rather are manifested as aberrant brain circuit structure or function [2–6].

Numerous neuroimaging studies suggest that specifically disrupted connectivity within the cortico-limbic system which consists of the amygdala, hippocampus, anterior cingulate cortex (ACC) and dorsolateral prefrontal cortex (DLPFC) [7] plays an important role in the pathogenesis of depression, presumably due in part to its importance in emotion generation and regulation processes [8–10]. It is also known that the hippocampus and amygdala subcortical limbic structures are imperative in depression [11–13]. According to both animal and human studies both the amygdala and hippocampus comprise anatomically and functionally discrete subfields [14–18]. The hippocampus is comprised of the dentate gyrus (DG), cornu ammonis (CA) 1–4, and subiculum [19]. The amygdala is known to be comprised of multiple nuclei which exhibit unique connectivity and molecular profiles [16, 19, 20]. Postmortem and animal studies indicate that depression is linked to morphological changes in the CA hippocampus subfields [21] and in the central and basolateral amygdala nuclei [22–26]. However, due to past technical constraints such as MRI spatial resolution and time-consuming manual tracing of the small subregion, in vivo studies considering the amygdala or hippocampus in depression have often treated them as single unified structures in analyses.

The emergence of automated segmentation techniques allowed for a feasibile and much improved investigation of subcortical substructures. There have been several studies on hippocampal subfields volumetrics in MDD, both manually and automated segmentation, some of which have reported reduced volumes in the dentate gyrus and CA (mainly CA3) [27–30], while others have found no significant differences between MDD subjects and healthy controls (HC) [31–33]. A previous study examining hippocampus subfields and amygdala nuclei volumetric differences between MDD and HC found no significant group differences, yet, reduced volumes of the amygdala’s right lateral nucleus (LA), left cortical nucleus (CoA), left accessory basal nucleus (ABA) and bilateral corticoamygdaloid transition area (CAT) were associated with depression severity within the MDD group [34]. However, in-vivo structural connectivity characterizations of the amygdala or hippocampus subregions in humans are challenging due to technical barriers in discerning these nuclei with the relatively low spatial resolution of diffusion-weighted imaging.

Ultra-high field 7-Tesla (7T) MRI allows imaging with higher signal-to-noise and contrast-to-noise ratios for improved spatial resolution [35]. Our recent ultra-high resolution 7T diffusion MRI studies on a subset of the current sample found reduced number of streamlines (i.e., white matter connections as reconstructed using probabilistic tractography) emerging from the hippocampal left DG subfield among MDD patients compared to HC [36], and that the right LA, basal nucleus (BA) and central (CeA) amygdala nuclei exhibited significantly increased connectivity to the rest of the brain, whereas the left medial nucleus (MeA) demonstrated significantly lower connectivity [37]. While these studies add to our understanding of the morphometric and structural connectivity differences of these small limbic structures that exist in MDD, they do not provide critical information about their role or hierarchy within the whole-brain network.

A network-based perspective is needed to study the topology or hierarchy of regions within the whole brain system. The mathematical field of graph theory offers a flexible way to model whole-brain connectivity, known as the connectome [38–40]. The structural brain network graph is composed of *nodes*, representing anatomical regions of interest (ROIs), and *edges*, representing connections. Local nodal features (e.g., node strength, betweenness centrality, and clustering coefficient) are used to depict the network regions’ hierarchy in the network and identify hubs that are critical for an efficient information flow [39]. To account for the hippocampus and amygdala small subregions function in the network we apply a graph theory approach and to our knowledge, no such studies have been performed.

Here, we examined the role of the small amygdala and hippocampus subregions in the whole-brain neural network and their topological features in relation to depression symptoms among MDD patients and HC using ultra-high field 7T MRI. Utilizing the 7T MRI high spatial resolution advantage, data-driven graph theory structural connectomic analysis was implemented to test whether local network hierarchies of amygdala and hippocampus subregions differentiate MDD and HC. We examined three common local centrality features; (1) node strength, which quantifies the node’s connectedness in the network and is defined by the sum of weights of connections to the node. Higher node strength indicates a strong and direct influence on the other nodes in the network. In brain networks, nodes with high node strength are referred to as network hubs, and are thought to be critical for general information transmission and system-level computing, potentially making them good therapeutic targets for network-level modulation [39]; betweenness, which quantifies the node’s involvement in information flow across the network and defined as the number of shortest paths that traverse a given node. A node with a high betweenness centrality is more likely to act as an intermediary in the transmission of information between other nodes or even clusters of nodes in the network [41]. A node with higher betweenness centrality would have more control over the network since more information passes through that node; and (3) clustering coefficient, which quantifies the node’s connectedness to its local neighbors by calculating the probability that two nodes connected to a given node are also connected with each other (the node’s tendency to be embedded in a cluster). A node with a high clustering coefficient indicates its local cohesiveness and a high tendency to be embedded in or to form a cluster. A higher local clustering coefficient also indicates the network’s local robustness to the node’s removal or failure [42]. Thus, if a node’s neighbors are also highly connected with each other, removing that node will not greatly influence its neighbors’ ability to communicate with each other. We hypothesized that the MDD group will show aberrant limbic subregion structural network hierarchy compared to HC. We specifically hypothesized that the hippocampus dentate gyrus and CA 3 subfields and the CeA and BA amygdala nuclei will exhibit altered hierarchy in the brain network in relation to depression symptoms.

## Methods and materials

### Participants

Participants included 38 MDD patients (22 males, 16 females, mean age: 37.24 ± 11.45) and 40 HC (26 males, 14 females, mean age: 37.15 ± 10.40) age and gender-matched (*p* = 0.97 and *p* = 0.52, respectively). The demographic and clinical variables are presented in Table 1. All subjects were recruited at the Depression and Anxiety Center for Discovery and Treatment (DAC) at Icahn School of Medicine at Mount Sinai. All participants underwent the Structured Clinical Interview for DSM-V Axis Disorders (SCID-V) by a trained rater to determine any current or lifetime psychiatric disorder [43]. As the power analysis using G Power software [44] indicated that we needed a sample of at least 78 participants to detect a medium to large effect size (Cohen’s *d* = 0.65, *α* = 0.05, 1−*β* = 0.8) to conduct a two-sample t-test. Subjects were excluded if they had an unstable medical illness (i.e., a significant, active medical condition that requires treatment), history of neurological disease, history of schizophrenia or other psychotic disorder, neurodevelopmental/ neurocognitive disorder, substance use disorder within the past 2 years, any contraindications to MRI, or positive urine toxicology on the day of the scan. HC subjects were free from any current or lifetime psychiatric disorder. All participants were free of antidepressant medication or other psychotropic medication for at least 4 weeks (8 weeks for fluoxetine) prior to data collection. Inclusion criteria for MDD subjects included having MDD as their primary presenting problem and being in a current major depressive episode. In all subjects, depressive symptom severity was measured by a clinician with the Montgomery–Åsberg Depression Rating Scale (MADRS) [45], and subjective depression symptoms were assessed by the Quick Inventory of Depressive Symptomatology, Self-Report (QIDS-SR) [46]. All data were collected under Institutional Review Board (IRB) approved written informed consent and participants were compensated for their time.

**Table 1:** Demographic and clinical characteristics.

	MDD (*n* = 38)	HC (*n* = 40)	Statistic *χ*^*2*^/*t* (df)	*p-*value
Male (frequency, %)	22, 57.89%	26, 65%	0.42	0.52
Age, years (mean ± SD)	37.24 ± 11.45	37.15 ± 10.40	0.035 (76)	0.97
Age at first episode (mean ± SD)	20.45 ± 11.67	–	–	–
Years since first episode (mean ± SD)	16.79 ± 10.34	–	–	–
Number of episodes (mean ± SD)	4.40 ± 5.44 (35)	–	–	–
Duration of current episode, months (mean ± SD)	55.69 ± 71.28	–	–	–
Recurrent MDD (frequency, %)	27, 71.05%	–	–	–
Current PPD (frequency, %)	18, 47.37%	–	–	–
MADRS	29.34 ± 5.16	0.62 ± 1.18	33.88 (75)	2.16E−46*
QIDS-SR	14.06 ± 4.32 (36)	1.16 ± 1.79 (34)	16.72 (70)	1.35E−25*

*MDD* major depressive disorder, *HC* healthy controls, *PPD* persistent depressive disorder, *MADRS* Montgomery Åsberg Depression Rating Scale, *QIDS-SR* Quick Inventory of Depressive Symptomatology Self-Report. *p < 0.05 for MDD group compared to healthy control group.

### MRI data acquisition

Data were acquired on a Siemens Magnetom 7T MRI scanner (Erlangen, Germany) with a 32-channel head coil (Nova Medical, Wilmington, MA). Each imaging session included the acquisition of an anatomical scan using a twice magnetization-prepared rapid gradient echo (MP2RAGE) sequence for improved T1-weighted contrast and spatial resolution [47], with the following parameters: 0.7 mm isotropic resolution, 240 slices, TR/TE = 6000/3.62 ms, field of view (FOV) = 240 × 320, flip angle (FA) = 0 and 5°, bandwidth = 300. A coronal-oblique T2-weighted turbo spin-echo (T2-TSE) with the following parameters: resolution = 0.43 mm × 0.43 mm × 2.0 mm, 66 slices, TR/TE = 9000/69 ms, FOV = 816 mm × 1024, FA = 150°, bandwidth = 279. Lastly, a high-angular-resolved diffusion-weighted imaging (HARDI) sequence was acquired: *b* = 1500 s/mm^2^, 132 slices, TR/TE = 7200/67.6 ms, FOV = 210 mm × 210 mm, resolution = 1.05 mm isotropic, FA = 90°, and number of gradient directions was 64, with 5 *b* = 0 s/mm2. The 5 b = 0 acquisitions were interleaved during the acquisition to correct for artifacts at time points 0.0, 115.2, 223.2, 338.4, and 453.6 s. Two diffusion MRI reverse-direction scans were acquired to correct gradient distortions.

### Anatomical data processing

**Fig. 1:**
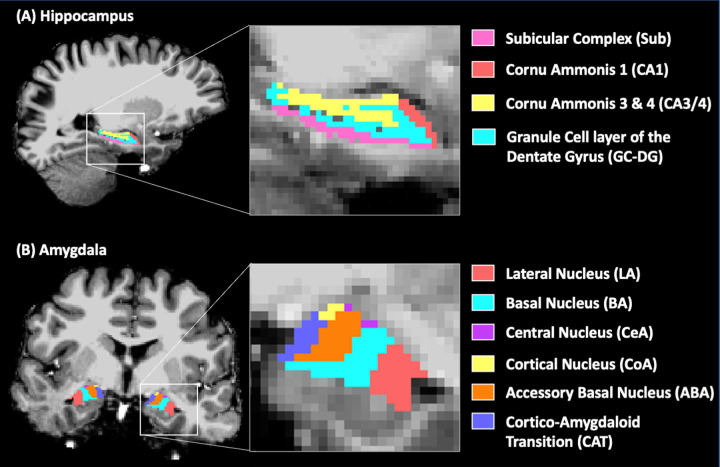
Hippocampus and amygdala subregion segmentation. A single-subject hippocampus (**A**) and amygdala (**B**) subregions FreeSurfer segmentation overlaid on the T1-weighted image.

T1-weighted images were preprocessed using the FreeSurfer (http://freesurfer.net) version 6.0 recon-all pipeline, nonparametric nonuniform intensity correction, intensity normalization, skull stripping, and neck removal, automatic segmentation, and parcellation steps [48]. Each subject’s anatomical brain image was segmented into the Desikan–Killiany Atlas [49] and subcortical brain regions. Hippocampus and amygdala segmentation was carried out in FreeSurfer development version 6.0 using the T1-weighted and coronal-oblique T2-TSE high-resolution images [34]. The hippocampus was segmented into the following subregions: presubiculum, subiculum, parasubiculum, CA1, CA3, CA4, the granule cell layer of the dentate gyrus (GC-DG), the molecular layer of the dentate gyrus, and the hippocampal-amygdala transition. The amygdala was segmented into the lateral (LA), basal (BA), accessory basal (ABA), cortical (CoA), medial (MeA), and central (CeA) nuclei and the corticoamygdaloid transition (CAT) area (Fig. 1B). All FreeSurfer outputs were manually inspected for segmentation quality, accuracy, and correct co-registration during the analysis. Hippocampus subfields were combined into CA1, CA3/4, the subicular complex (pre-, para- and subiculum), and the GC-DG (granule cell layer and molecular layer) to ensure subregions were large enough for accurate quantification (Fig. 1A). Due to the small volumes of the amygdala MeA, the authors chose to exclude this region from the analysis. All subregion volumes were quantified as the number of voxels within the diffusion-weighted image and compared between the MDD and HC using 2 sample t-tests. The whole-brain segmentation was combined with the hippocampus and amygdala subregions (a total of 98 ROIs) into a single brain parcellation image in the subject’s native space.

### Diffusion data processing

Diffusion data were preprocessed and denoised using MRtrix phase-reversed processing (https://mrtrix.readthedocs.io/en/latest/index.html). B1 field inhomogeneity correction was performed for the diffusion images [50]. Fiber orientation distributions (FODs) were estimated from the diffusion data using spherical deconvolution [50], and the diffusion tensor was calculated using iteratively reweighted linear least squares estimator [51]. The T1-weighted and the brain segmentation image (combined with hippocampus and amygdala subregions) were coregistered to the diffusion space using SPM12 nearest-neighbor interpolation. MRtrix software was used to carry out whole-brain probabilistic tractography [52]. Streamlines were thresholded using a FOD amplitude cutoff of 0.1. The spherical deconvolution (SIFT2) algorithm was applied to all tracts to eliminate spurious streamlines that were unlikely to be physically accurate [53]. Lastly, the streamlines count between all ROIs were extracted, creating the pairwise structural connectivity matrix.

### Connectome analysis

**Fig. 2:**
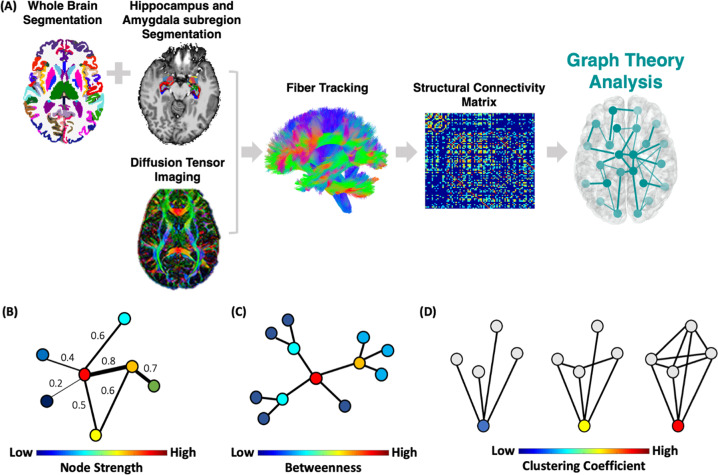
Connectome analysis procedure. **A** Each subject’s whole brain segmentation was combined with the hippocampus and amygdala subregion segmentation, constructing the nodes of the graph (a total of 98 ROIs). Network edges (i.e., links) were defined by the streamline count between all pairwise ROIs using probabilistic fiber tracking creating a structural connectivity matrix. A threshold of the top connections for each network was applied across a range from 0.1–0.3. We then examined the structural connectome local network centrality features; (**B**) node strength—the sum of weights of links connected to the brain region; (**C**) betweenness centrality—the number of shortest paths that traverse a given region; and **D** clustering coefficient, which quantifies how well a node’s neighbors are also connected to one another. Finally, for each local feature, the area under the curve for all network densities was used to provide a measure independent of single threshold selection.

To construct the brain structural connectome each of the 98 anatomical segmented ROIs represents a node in the graph and the network edges were defined by the streamline count between any pairwise ROIs derived from the diffusion MRI tractography matrix (Fig. 2A). We then used a sparsity threshold S, which retains S% of the top connections for each subject to ensure that the number of nodes and connections are matched across participants [6].

Using the Brain Connectivity Toolbox [54], we examined common local nodal centrality features; (1) *node strength*, which is the sum of weights of links connected to the node (Fig. 2B); (2) *betweenness centrality*, which is a measure of the number of shortest paths that traverse a given node (Fig. 2C); and (3) *clustering coefficient*, which quantifies how well a node’s neighbors are also connected to one another and is defined by the fraction of triangles around a node (Fig. 2D). We examined these local centrality features across a range of thresholds (10% < *S* < 30% in steps of 1%) [6]. We then calculated the area under the curve for each network feature, which provides a summarized measure independent of a single threshold selection [6].

### Statistical analysis

We conducted a between-group two-sample t-test for each region’s centrality measures. Age, gender, and region volume were treated as covariates in all analyses. For statistical significance permutation tests were performed conducting 1000 repetitions on shuffled datasets assessing the specificity of the t-test findings by comparing the observed *t*-value with the results of the randomized networks. The *p*-value of the bootstrapping test was defined as the fraction of the number of random cases which obtained t statistic values smaller than the observed finding. Finally, all results were corrected for multiple comparisons for a total of 98 tests (number of regions) using the false discovery rate (FDR) correction [55] (*q* < 0.05).

For the MDD group separately, we also conducted Spearman’s correlations to assess the association between the hippocampus and amygdala subregions centrality features and the depression symptoms as measured by the MADRS score. Partial correlation was used to control for age, gender, subregion volume, and previous antidepressant medication history (1 = had previous medication treatment, 0 = no past medication treatment) as covariates. The results were corrected for multiple comparisons using FDR (*q* < 0.05) where the hippocampus subregions were corrected for a total of 8 tests and amygdala subregions for a total of 12 (number of subregions).

## Results

The groups were matched for age and gender (see Table 1). Significant differences in MADRS (*t(df)=* 33.88 (75), *p* = 2.16E−46) and QIDS-SR (*t(df)* = 16.72 (70), *p* = 1.35E−25) were present (Table 1). Assumptions for normal distribution and equal variances were met for two-sided t-tests.

**Fig. 3:**
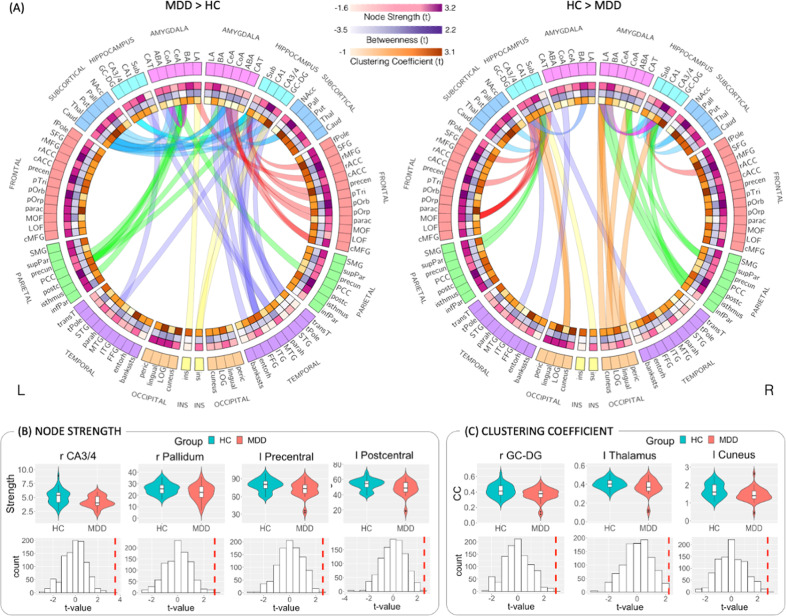
Connectome analysis results. **A** Connectograms of amygdala and hippocampus subregions hyper-connectivity (MDD > HC) and hypo-connectivity (HC > MDD). The ideograms (i.e., heatmap inner rings) represent the between-groups differences (t-values) in node strength (pink), betweenness (purple), and clustering coefficient (orange), the darker the tone the greater the group difference. All structural connectivity that are significantly different between the two groups at the *p* < 0.05 uncorrected level are plotted as edges. Each edge is color-coded according to the brain’s anatomical lobe. Amygdala and hippocampus subregions exhibit both hyper- and hypo-connectivity and inter- and intra-hemispheric connections. The connectogram visualization was created using Circos (http://circos.ca/). Violin plots and permutation tests histograms of the significant between-group differences (*p* < 0.05 FDR corrected) for the local network features of node strength (**B**) and clustering coefficients (**C**). The histograms represent the *t*-value results of 1000 permutations tests, and the vertical red line represents the true *t*-value comparing the MDD vs. HC.

**Fig. 4:**
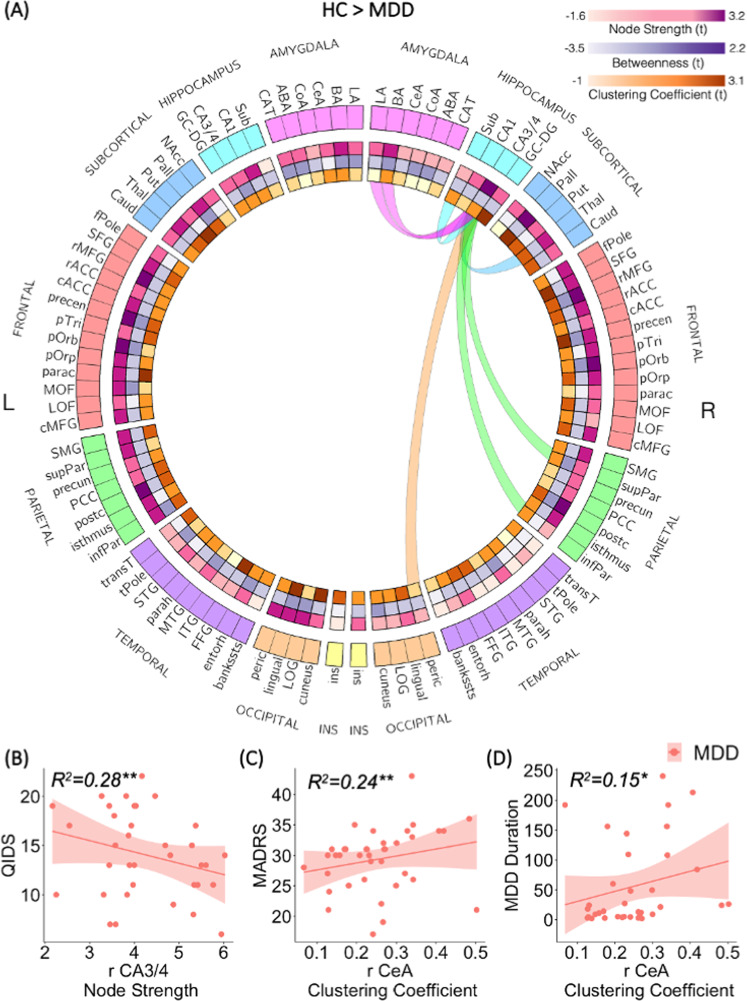
Hippocampus CA3/4 connectogram and association to clinical measures. **A** Compared to HC, the right hippocampus CA3/4 exhibited significantly decreased node strength (lower connectivity to the rest of the brain network) among the MDD group. Connectogram visualization of the right hippocampus CA3/4 reduced connectivity among the MDD group compared to HC. Each edge is color-coded according to the brain’s anatomical lobe. The connectogram visualization was created using Circos (http://circos.ca/). **B** Patients with greater depression symptoms as measured by the QIDS score exhibit lower right hippocampus CA3/4 node strength. **C** The Amygdala right CeA clustering coefficient feature exhibit a positive correlation to depression symptoms as measured by the MADRS score (**p < 0.05, FDR corrected) among the MDD group. Further exploration also found a correlation to depressive episode duration (*p < 0.05, uncorrected) (**D**).

To test our hypothesis regarding the relationship between network topology and depression, we investigated the difference in the local (e.g., node strength, betweenness centrality, and clustering coefficient) subregion network features (within the whole brain connectome) between MDD and HC (Fig. 3). We found that compared to HC, MDD patients exhibit decreased strength of the right hippocampus CA3/4 subfield (*t*(*df*) = 3.56 (71), *p* < 0.001, *Cohen’s d* = 0.73), right pallidum (*t*(*df*) = 2.83 (71), *p* < 0.001, *Cohen’s d* = 0.59), left precentral gyrus (*t*(*df*) = 2.55 (71), *p* < 0.001, *Cohen’s d* = 0.58) and left postcentral gyrus (*t*(*df*) = 2.58 (71), *p* < 0.001, *Cohen’s d* = 0.60), all FDR corrected (*q* < 0.05) controlled for age, gender, region’s volume and previous antidepressant medication history (Fig. 3B). In other words, these regions exhibit lower connectivity to the rest of the brain network among the MDD group compared to the HC group. As the focus of the analysis was to examine the role of the limbic subregions in the network, we conducted further specific exploration of the right hippocampus CA3/4 edges’ weights (number of streamlines connecting two regions). No significant edges weights differences between the MDD and HC groups were found after correcting for multiple comparisons. However, at an uncorrected threshold in a one-sided two-sample t-test, decreased number of streamlines was observed between the right hippocampus CA3/4 and the right thalamus, pericalcarine, inferior parietal, hippocampus subiculum subfield, and amygdala’s lateral and basal nuclei (MDD < HC; *p* < 0.05, uncorrected, Table S2 in the Supplementary and Fig. 4A).

Compared to HC, MDD patients also exhibit decreased clustering coefficient measures of the right hippocampus GC-DG (*t*(*df*) = 2.90 (71), *p* < 0.001, *Cohen’s d* = 0.61), left cuneus (*t*(*df*) = 2.77 (71), *p* < 0.001, *Cohen’s d* = 0.65) and left thalamus (*t*(*df*) = 2.22 (71), *p* < 0.001, *Cohen’s d* = 0.50), all FDR corrected (*q* < 0.05) (Fig. 3C), indicating these regions are less embedded in a cluster among patients. None of the betweenness centralities measures showed significant differences between the MDD and control groups after correcting for multiple comparisons.

In addition to controlling for the region’s volume, the volumes of the amygdala and hippocampal subregions within the diffusion image were compared between MDD and HC to determine whether any structural connectivity differences were being driven by differences in volume. There were no subregion volumes that significantly differed between MDD and HC (Supplementary S1).

Further investigation of the significant results association with depression symptoms among MDD patients, found reduced hippocampus CA3/4 node strength was significantly associated with greater self-reported depression symptoms as measured by the QIDS-SR score (*r* = −0.53, *p* < 0.0035, FDR corrected) (Fig. 4B).

In addition, we tested whether the network topology of only the limbic subregions is associated with depression symptoms as measured by the clinical MADRS and self-reported QIDS-SR scores as well as the depressive episode duration. The amygdala right central nucleus clustering coefficient showed a positive association with MADRS scores (*r* = 0.49, *p* < 0.004, FDR corrected) among the MDD group controlled for age, gender, subregion volume, and previous antidepressant medication history (Fig. 4C). The right central nucleus clustering coefficient was also positively correlated with the depressive episode duration (*r* = 0.39, *p* < 0.03 uncorrected) (Fig. 4D).

## Discussion

Applying a data-driven connectomic approach with high-field 7-T diffusion MRI data, we investigated the role and topological architecture of minute structures of the limbic amygdala and hippocampus within the whole brain network in MDD patients compared to HC and their association with depression symptoms. Analysis of the network hierarchy according to node strength showed reduced strength (i.e., reduced connectivity to the rest of the brain network) of the right hippocampus CA3/4 among MDD patients compared to HC (Fig. 3B). Decreased hippocampus CA3/4 connectivity was also associated with greater self-reported depression symptoms (Fig. 4). This reduced hippocampus CA3/4 overall connectivity might be driven by a trend indicating a reduced number of streamlines both internally between the hippocampus right CA3/4 and right subiculum as well as externally with other limbic structures including the right thalamus and amygdala’s lateral and basal nuclei (Fig. 4A). Analysis of the network hierarchy according to nodal clustering coefficients found that compared to HC, MDD patients’ hippocampus GC-DG subregion exhibit a reduced tendency to be embedded in a cluster (Fig. 3C). In contrast, a stronger clustering coefficient of the right amygdala central nucleus was associated with higher depression symptoms levels and depressive episode duration among MDD patients (Fig. 4C, D). Together, these results indicate specific subregions altered involvement in the whole brain connectome as related to depression.

The hippocampus dentate gyrus and CA 3 are the most implicated subfields in relation to depression [56]. Here, both (i.e., GC-DG and CA3/4) were the only subregions that exhibit a significantly altered connectivity pattern among MDD compared to HC. The hippocampus CA3/4 showed decreased overall connectivity to the rest of the brain network and the dentate gyrus exhibited a reduced tendency to be embedded in a cluster among MDD. In a previous study, we found a reduced overall number of streamlines emerging from the hippocampal left GC-DG subfield among MDD patients compared to HC [36], whereas here we did not observe a significant reduction in GC-DG strength. However, the right dentate gyrus did exhibit a reduced tendency to be embedded in a cluster among MDD. This result might indicate the network’s lack of endurance (or compensation) in case of a hippocampus GC-DG failure. Preclinical studies have shown that chronic stress or overexposure to glucocorticoids (i.e., stress hormones cortisol in humans, corticosterone in rodents) leads to atrophy in the hippocampus dentate gyrus, CA, and particularly of the pyramidal neurons of CA3 [57]. Other studies also showed that antidepressant treatments can prevent and reverse this atrophy in CA3 [56, 58, 59]. A postmortem human study [21] found significant decreased pyramidal neuron soma size in all CA hippocampus subfields (CA1–CA4) among MDD patients compared to HC and a neuroimaging anatomical study found that patients with MDD showed a bilateral pattern of volume reduction in all CA subfields [60]. Here we show that even when controlling for hippocampus CA3/4 subregion volume, its role in the whole brain network is significantly reduced among nonmedicated MDD patients exhibiting less connectivity to the rest of the brain.

The hippocampus is part of the limbic system and is known to be involved in the formation of episodic and declarative memory [61]. Specifically, the hippocampus connectivity to the thalamus through the mammillary bodies and fornix is known to be crucial for episodic event memory [62, 63]. Impaired overall connectivity of the hippocampus CA3/4 subregion and specifically the trend in its reduced connectivity with the thalamus (Fig. 4A) among depressed people are in line with previous studies showing impaired episodic memory and particularly autobiographical memory in depressed patients [64, 65]. In addition, our results also indicate a trend of reduced connectivity of the right hippocampus CA3/4 with the ipsilateral BA and LA amygdala nuclei (Fig. 4A), which may cause emotional memory biases in depression. However, these results must be interpreted with caution as they are uncorrected for multiple comparisons.

While the focus of the current analysis was to examine the role of hippocampus and amygdala subregions in the whole brain network, several other brain regions exhibited significantly altered connectivity patterns among MDD compared to HC. The right pallidum, left precentral, and postcentral gyri showed decreased node strength (i.e., reduced connectivity to the whole brain network regions); the left cuneus and thalamus showed decreased clustering coefficient (i.e., reduced tendency to cluster). Alterations in the structure and function of each of these regions have been linked to depression in several prior studies [66–70]. According to meta-analyses, MDD patients showed decreased pallidum, thalamus [67], and precentral gyrus [69] volumes, increased cuneus volume [70], and decreased activation in the precentral and postcentral gyri [68]. Here we show that when controlling for the region’s volumes, their role in the whole brain network is significantly reduced among MDD patients exhibiting less connectivity to the rest of the brain or less connectedness to clusters.

Exploration of disease severity association with the subregion’s connectome features found the amygdala CeA subregion clustering coefficient to be highly correlated with depression symptoms scores (Fig. 4). A higher clustering coefficient indicates that this region’s neighbors are also highly connected between them (highly clustered). Previous studies have shown that the amygdala CeA is highly connected to key cortical and subcortical brain regions functioning as a hub mediating various aspects of stress response as well as of fear and anxiety [71–76]. To our knowledge, this is the first in vivo human study showing the association between CeA hubness (centrality in the network) and depression severity.

The main limitations of this study are the potential effect of partial voluming of the small limbic substructure and the limited sample size. To minimize the partial volume, we excluded very small hippocampal subfields and amygdaloid nuclei that were not consistently accurately delineated on T2-TSE and T1 images [36]. Additionally, we merged several smaller constituent regions into their larger subfield (dentate gyrus and subicular complex). Thus, the final limbic subregions used in the current analysis were large enough to both be segmented using ultra-high submillimeter resolution T1 and T2-TSE imaging and accurately visualized to conduct tractography using the 1.05 mm isotropic diffusion imaging. Another major limitation of this study is our limited accountability for prior medication treatment effects among MDD patients. Though no participants were taking antidepressant medication at the time and 4 weeks prior to clinical evaluation and scanning, most of our patients have been receiving drug therapies in the past. To account for any confounding effects, we statistically controlled for prior medication treatment. Future prospective drug therapy studies could shed a light on the effect of medication on the limbic substructures connectomic.

To conclude, our findings add to existing research by inspecting the whole-brain network hierarchy of small limbic subregions and identifying the hippocampus CA3/4 and GC-DG subfields as critical nodes in MDD pathophysiology and the amygdala CeA nucleus as predictive of depression symptoms levels. Compared to previous studies, this study was conducted on an ultra-high field 7 T dataset which offers considerable advantage of improved quality of signal and thus allows for more precise segmentation and structural connectivity analysis. We believe that these findings may advance our knowledge regarding the underpinning mechanisms of depression and its relevance to potential treatments.

## Supplementary information

Supplementary

## Data Availability

The code that supports the findings of the present study is available from the corresponding author upon request. The data and code sharing adopted by the authors comply with the requirements of the funding institute and with institutional ethics approval.

## References

[1] Kessler RC, Chiu WT, Demler O, Merikangas KR, Walters EE (2005). Prevalence, severity, and comorbidity of 12-month DSM-IV disorders in the National Comorbidity Survey Replication. Arch Gen Psychiatry.

[2] Etkin A, Büchel C, Gross JJ (2015). The neural bases of emotion regulation. Nat Rev Neurosci.

[3] Menon V (2011). Large-scale brain networks and psychopathology: A unifying triple network model. Trends Cogn Sci.

[4] Sylvester C, Corbetta M, Raichle M, Rodebaugh T, Schlaggar B, Sheline Y (2012). Functional network dysfunction in anxiety and anxiety disorders. Trends Neurosci.

[5] Repple J, Mauritz M, Meinert S, de Lange SC, Grotegerd D, Opel N (2020). Severity of current depression and remission status are associated with structural connectome alterations in major depressive disorder. Mol Psychiatry.

[6] Korgaonkar MS, Fornito A, Williams LM, Grieve SM (2014). Abnormal structural networks characterize major depressive disorder: A connectome analysis. Biol Psychiatry.

[7] Rajmohan V, Mohandas E (2007). The limbic system. Indian J Psychiatry.

[8] Johnstone T, Van Reekum CM, Urry HL, Kalin NH, Davidson RJ (2007). Failure to regulate: Counterproductive recruitment of top-down prefrontal-subcortical circuitry in major depression. J Neurosci.

[9] Martin EI, Ressler KJ, Binder E, Nemeroff CB (2009). The neurobiology of anxiety disorders: brain imaging, genetics, and psychoneuroendocrinology. Psychiatr Clin North Am.

[10] Price JL, Drevets WC (2010). Neurocircuitry of mood disorders. Neuropsychopharmacology..

[11] Drevets WC (2003). Neuroimaging abnormalities in the amygdala in mood disorders. Ann N. Y Acad Sci.

[12] Videbech P, Ravnkilde B (2004). Hippocampal volume and depression: A meta-analysis of MRI studies. Am J Psychiatry.

[13] Engin E, Treit D (2007). The role of hippocampus in anxiety: Intracerebral infusion studies. Behavioural Pharmacol.

[14] Balderston NL, Schultz DH, Hopkins L, Helmstetter FJ (2015). Functionally distinct amygdala subregions identified using DTI and high-resolution fMRI. Soc Cogn Affect Neurosci.

[15] Hrybouski S, Aghamohammadi-Sereshki A, Madan CR, Shafer AT, Baron CA, Seres P (2016). Amygdala subnuclei response and connectivity during emotional processing. NeuroImage..

[16] Chang SWC, Fagan NA, Toda K, Utevsky AV, Pearson JM, Platt ML (2015). Neural mechanisms of social decision-making in the primate amygdala. Proc Natl Acad Sci USA.

[17] Oler JA, Tromp DP, Fox AS, Kovner R, Davidson RJ, Alexander AL (2017). Connectivity between the central nucleus of the amygdala and the bed nucleus of the stria terminalis in the non-human primate: Neuronal tract tracing and developmental neuroimaging studies. Brain Struct Funct.

[18] Xu Y, Day TA, Buller K (1999). The central amygdala modulates hypothalamic–pituitary–adrenal axis responses to systemic interleukin-1β administration. Neuroscience..

[19] Ding S-L, Royall JJ, Sunkin SM, Ng L, Facer BAC, Lesnar P (2017). Comprehensive cellular-resolution atlas of the adult human brain. J Comp Neurol.

[20] Cho YT, Ernst M, Fudge JL (2013). Cortico–amygdala–striatal circuits are organized as hierarchical subsystems through the primate amygdala. J Neurosci.

[21] Stockmeier CA, Mahajan GJ, Konick LC, Overholser JC, Jurjus GJ, Meltzer HY (2004). Cellular changes in the postmortem hippocampus in major depression. Biol Psychiatry.

[22] Rubinow MJ, Mahajan G, May W, Overholser JC, Jurjus GJ, Dieter L (2016). Basolateral amygdala volume and cell numbers in major depressive disorder: A postmortem stereological study. Brain Struct Funct.

[23] Gonçalves L, Silva R, Pinto-Ribeiro F, Pêgo JM, Bessa JM, Pertovaara A (2008). Neuropathic pain is associated with depressive behaviour and induces neuroplasticity in the amygdala of the rat. Exp Neurol.

[24] Joshi SH, Espinoza RT, Pirnia T, Shi J, Wang Y, Ayers B (2016). Structural plasticity of the hippocampus and amygdala induced by electroconvulsive therapy in major depression. Biol Psychiatry.

[25] Seno MDJ, Assis DV, Gouveia F, Antunes GF, Kuroki M, Oliveira CC (2018). The critical role of amygdala subnuclei in nociceptive and depressive-like behaviors in peripheral neuropathy. Sci Rep.

[26] Faria V, Appel L, Åhs F, Linnman C, Pissiota A, Frans Ö (2012). Amygdala subregions tied to SSRI and placebo response in patients with social anxiety disorder. Neuropsychopharmacology..

[27] Maller JJ, Broadhouse K, Rush AJ, Gordon E, Koslow S, Grieve SM (2018). Increased hippocampal tail volume predicts depression status and remission to anti-depressant medications in major depression. Mol Psychiatry.

[28] Huang Y, Coupland NJ, Lebel RM, Carter R, Seres P, Wilman AH (2013). Structural changes in hippocampal subfields in major depressive disorder: A high-field magnetic resonance imaging study. Biol Psychiatry.

[29] Treadway MT, Waskom ML, Dillon DG, Holmes AJ, Park MTM, Chakravarty MM (2015). Illness progression, recent stress, and morphometry of hippocampal subfields and medial prefrontal cortex in major depression. Biol Psychiatry.

[30] Han K-M, Won E, Sim Y, Tae W-S (2016). Hippocampal subfield analysis in medication-naive female patients with major depressive disorder. J Affect Disord.

[31] Travis SG, Coupland NJ, Hegadoren K, Silverstone PH, Huang Y, Carter R (2016). Effects of cortisol on hippocampal subfields volumes and memory performance in healthy control subjects and patients with major depressive disorder. J Affect Disord.

[32] Na K-S, Chang HS, Won E, Han K-M, Choi S, Tae WS (2014). Association between glucocorticoid receptor methylation and hippocampal subfields in major depressive disorder. PLoS One.

[33] Tannous J, Godlewska BR, Tirumalaraju V, Soares JC, Cowen PJ, Selvaraj S (2020). Stress, inflammation and hippocampal subfields in depression: A 7 Tesla MRI Study. Transl Psychiatry.

[34] Brown SSG, Rutland JW, Verma G, Feldman RE, Alper J, Schneider M (2019). Structural MRI at 7T reveals amygdala nuclei and hippocampal subfield volumetric association with major depressive disorder symptom severity. Sci Rep.

[35] Vu TA, Jamison K, Glasser MF, Smith SM, Coalson T, Moeller S (2017). Tradeoffs in pushing the spatial resolution of fMRI for the 7T Human Connectome Project. NeuroImage..

[36] Rutland JW, Brown S, Verma G, Feldman RE, Sharma H, Markowitz M (2019). Hippocampal subfield-specific connectivity findings in major depressive disorder: A 7 Tesla diffusion MRI study. J Psychiatr Res.

[37] Brown SS, Rutland JW, Verma G, Feldman RE, Schneider M, Delman BN (2020). Ultra-high-resolution imaging of amygdala subnuclei structural connectivity in major depressive disorder. Biol Psychiatry: Cogn Neurosci Neuroimaging.

[38] Bullmore E, Sporns O (2009). Complex brain networks: Graph theoretical analysis of structural and functional systems. Nat Rev Neurosci.

[39] Sporns O. Networks of the brain. Cambridge, MA, US: MIT Press; 2010.

[40] Bassett DS, Bullmore E, Verchinski BA, Mattay VS, Weinberger DR, Meyer-Lindenberg A (2008). Hierarchical organization of human cortical networks in health and schizophrenia. J Neurosci.

[41] Bloch F, Jackson MO, Tebaldi P. Centrality measures in networks. arXiv preprint arXiv:1608.05845. 2016.

[42] Latora V, Nicosia V, Panzarasa P (2013). Social cohesion, structural holes, and a tale of two measures. J Stat Phys.

[43] First M, Williams J, Karg R, Spitzer R. Structured clinical interview for DSM-5—Research version (SCID-5 for DSM-5, research version; SCID-5-RV). Arlington, VA: American Psychiatric Association; 2015.

[44] Faul F, Erdfelder E, Lang A-G, Buchner A (2007). G* Power 3: A flexible statistical power analysis program for the social, behavioral, and biomedical sciences. Behav Res Methods.

[45] Montgomery SA, Asberg M (1979). A new depression scale designed to be sensitive to change. Br J Psychiatry.

[46] Rush AJ, Trivedi MH, Ibrahim HM, Carmody TJ, Arnow B, Klein DN (2003). The 16-Item Quick Inventory of Depressive Symptomatology (QIDS), clinician rating (QIDS-C), and self-report (QIDS-SR): A psychometric evaluation in patients with chronic major depression. Biol Psychiatry.

[47] Marques JP, Kober T, Krueger G, van der Zwaag W, Van de Moortele P-F, Gruetter R (2010). MP2RAGE, a self bias-field corrected sequence for improved segmentation and T1-mapping at high field. Neuroimage..

[48] Fischl B, Salat DH, Busa E, Albert M, Dieterich M, Haselgrove C (2002). Whole brain segmentation: Automated labeling of neuroanatomical structures in the human brain. Neuron..

[49] Desikan RS, Segonne F, Fischl B, Quinn BT, Dickerson BC, Blacker D (2006). An automated labeling system for subdividing the human cerebral cortex on MRI scans into gyral based regions of interest. Neuroimage..

[50] Tustison NJ, Avants BB, Cook PA, Zheng Y, Egan A, Yushkevich PA (2010). N4ITK: Improved N3 bias correction. IEEE Trans Med Imaging.

[51] Veraart J, Sijbers J, Sunaert S, Leemans A, Jeurissen B (2013). Weighted linear least squares estimation of diffusion MRI parameters: Strengths, limitations, and pitfalls. Neuroimage..

[52] Smith RE, Tournier J-D, Calamante F, Connelly A (2012). Anatomically-constrained tractography: Improved diffusion MRI streamlines tractography through effective use of anatomical information. Neuroimage..

[53] Smith RE, Tournier J-D, Calamante F, Connelly A (2015). SIFT2: Enabling dense quantitative assessment of brain white matter connectivity using streamlines tractography. Neuroimage..

[54] Rubinov M, Sporns O (2010). Complex network measures of brain connectivity: Uses and interpretations. Neuroimage..

[55] Benjamini Y, Hochberg Y (1995). Controlling the false discovery rate: A practical and powerful approach to multiple testing. J R Stat Soc Ser B.

[56] Sapolsky RM (2000). Glucocorticoids and hippocampal atrophy in neuropsychiatric disorders. Arch Gen Psychiatry.

[57] Adam Samuels B, Leonardo ED, Hen R (2015). Hippocampal subfields and major depressive disorder. Biol Psychiatry.

[58] Czéh B, Lucassen PJ (2007). What causes the hippocampal volume decrease in depression?. Eur Arch Psychiatry Clin Neurosci.

[59] Conrad CD (2008). Chronic stress-induced hippocampal vulnerability: The glucocorticoid vulnerability hypothesis. Rev Neurosci.

[60] Roddy DW, Farrell C, Doolin K, Roman E, Tozzi L, Frodl T (2019). The hippocampus in depression: More than the sum of its parts? Advanced hippocampal substructure segmentation in depression. Biol Psychiatry.

[61] Scoville WB, Milner B (1957). Loss of recent memory after bilateral hippocampal lesions. J Neurol, Neurosurg, Psychiatry.

[62] Aggleton JP, O’Mara SM, Vann SD, Wright NF, Tsanov M, Erichsen JT (2010). Hippocampal–anterior thalamic pathways for memory: Uncovering a network of direct and indirect actions. Eur J Neurosci.

[63] Burgess N, Maguire EA, O’Keefe J (2002). The human hippocampus and spatial and episodic memory. Neuron..

[64] Söderlund H, Moscovitch M, Kumar N, Daskalakis ZJ, Flint A, Herrmann N (2014). Autobiographical episodic memory in major depressive disorder. J Abnorm Psychol.

[65] Dillon DG, Pizzagalli DA (2018). Mechanisms of memory disruption in depression. Trends Neurosci.

[66] Taki Y, Kinomura S, Awata S, Inoue K, Sato K, Ito H (2005). Male elderly subthreshold depression patients have smaller volume of medial part of prefrontal cortex and precentral gyrus compared with age-matched normal subjects: A voxel-based morphometry. J Affect Disord.

[67] Espinoza Oyarce DA, Shaw ME, Alateeq K, Cherbuin N (2020). Volumetric brain differences in clinical depression in association with anxiety: A systematic review with meta-analysis. J Psychiatry Neurosci.

[68] Delaveau P, Jabourian M, Lemogne C, Guionnet S, Bergouignan L, Fossati P (2011). Brain effects of antidepressants in major depression: A meta-analysis of emotional processing studies. J Affect Disord.

[69] Bora E, Fornito A, Pantelis C, Yücel M (2012). Gray matter abnormalities in major depressive disorder: A meta-analysis of voxel based morphometry studies. J Affect Disord.

[70] Peng W, Chen Z, Yin L, Jia Z, Gong Q (2016). Essential brain structural alterations in major depressive disorder: A voxel-wise meta-analysis on first episode, medication-naive patients. J Affect Disord.

[71] Kalin NH, Shelton SE, Davidson RJ (2004). The role of the central nucleus of the amygdala in mediating fear and anxiety in the primate. J Neurosci.

[72] Amaral DG, Price JL, Pitkanen A, Carmichael ST. Anatomical organization of the primate amygdaloid complex. In The Amygdala: Neurobiological Aspects of Emotion, Memory, and Mental Dysfunction. New York, NY, US: Wiley-Liss; 1992. p. 1–66.

[73] Oler JA, Fox AS, Shackman AJ, Kalin NH. Living without an amygdala. New York, NY, US: The Guilford Press; 2016. P. 218–51.

[74] Gilpin NW, Herman MA, Roberto M (2015). The central amygdala as an integrative hub for anxiety and alcohol use disorders. Biol Psychiatry.

[75] LeDoux J, Iwata J, Cicchetti P, Reis D (1988). Different projections of the central amygdaloid nucleus mediate autonomic and behavioral correlates of conditioned fear. J Neurosci.

[76] Kinreich S, Intrator N, Hendler T (2011). Functional cliques in the amygdala and related brain networks driven by fear assessment acquired during movie viewing. Brain Connectivity.

