# Supplementary material for: Altered hippocampus and amygdala subregion connectome hierarchy in major depressive disorder
Source: Transl Psychiatry. 2022 May 19;12:209. doi: 10.1038/s41398-022-01976-0 (PMC9120054; doi:10.1038/s41398-022-01976-0)
Supplement: Supplementary file 1 — Supplementary [file 41398_2022_1976_MOESM1_ESM.docx]

**Altered Hippocampus and Amygdala** **Subregion Connectome Hierarchy in Major Depressive Disorder**

**Supplementary**

Yael Jacob, Laurel S Morris, Gaurav Verma, Sarah Rutter, Priti Balchandani, James W Murrough

**Table S1. Subregion’s volumes**

| ***Subregion*** | ***MDD***  ***(Mean ± SD)*** | ***HC***  ***(Mean ± SD)*** | ***t-value*** | ***p-value*** |
| --- | --- | --- | --- | --- |
| l Sub | 551.68 ± 113.67 | 537.95 ± 77.20 | -0.61 | 0.54 |
| r Sub | 541.33 ± 71.55 | 536.76 ± 86.57 | -0.25 | 0.80 |
| l CA1 | 517.63 ± 85.02 | 492.27 ± 77.67 | -1.36 | 0.18 |
| r CA1 | 531.93 ± 64.94 | 518.65 ± 79.59 | -0.80 | 0.42 |
| l CA3/4 | 369.15 ± 58.85 | 354.43 ± 57.18 | -1.11 | 0.27 |
| r CA3/4 | 360.18 ± 48.63 | 345.41 ± 52.45 | -1.28 | 0.20 |
| l GC-DG | 708.33 ± 115.61 | 707.68 ± 107.67 | -0.03 | 0.98 |
| r GC-DG | 697.68 ± 77.72 | 687.30 ± 95.10 | -0.53 | 0.60 |
| l LA | 453.00 ± 71.21 | 453.95 ± 69.76 | 0.06 | 0.95 |
| r LA | 503.45 ± 62.64 | 468.78 ± 69.71 | -2.30 | 0.02 |
| l BA | 365.28 ± 51.27 | 354.76 ± 39.85 | -1.00 | 0.32 |
| r BA | 363.20 ± 39.95 | 344.27 ± 54.33 | -1.75 | 0.08 |
| l CeA | 35.65 ± 35.97 | 30.89 ± 7.15 | -0.79 | 0.43 |
| r CeA | 29.40 ± 7.79 | 28.73 ± 10.32 | -0.32 | 0.75 |
| l CoA | 18.18 ± 6.95 | 19.38 ± 8.59 | 0.68 | 0.50 |
| r CoA | 12.33 ± 3.38 | 12.78 ± 5.06 | 0.47 | 0.64 |
| l ABA | 194.48 ± 44.52 | 195.70 ± 28.13 | 0.14 | 0.89 |
| r ABA | 202.95 ± 27.09 | 203.76 ± 35.61 | 0.11 | 0.91 |
| l CAT | 126.18 ± 30.65 | 124.24 ± 19.40 | -0.33 | 0.74 |
| r CAT | 133.68 ± 17.37 | 132.19 ± 21.89 | -0.33 | 0.74 |

Between group statistical analysis results for amygdala and hippocampal subregions volumes as assessed by the number of voxles within the diffusion image. (Sub, Subicular complex; CA, cornu ammonis; GC-DG, granule cell layer of the dentate gyrus; LA, lateral nucleus; BA, basal nucleus; CeA, central nucleus; CoA, cortical nucleus; ABA, accessory basal; CAT, corticoamygdaloid transition).


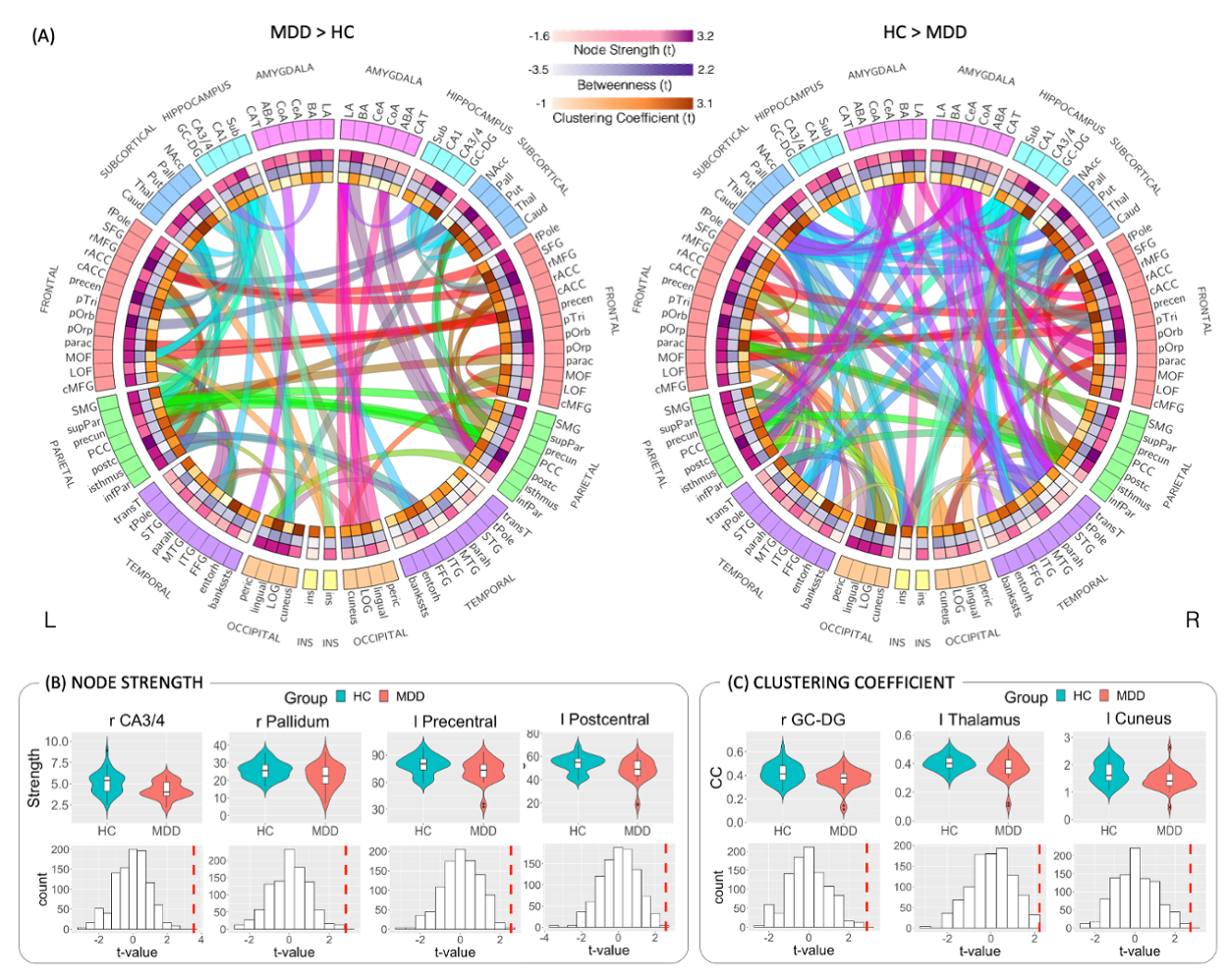


**Figure S1. (A)** Connectograms of whole brain hyper-connectivity (MDD > HC) and hypo-connectivity (HC > MDD). The ideograms (i.e., heatmap inner rings) represent the between groups differences (t-values) in node strength (pink), betweenness (purple) and clustering coefficient (orange), the darker the tone the greater the group difference. All structural connectivity’s that are significantly different between the two groups at the p < 0.05 uncorrected level are plotted as edges. Each edge is color-coded according to the brain anatomical lobe. Amygdala and hippocampus subregions exhibit both hyper- and hypo-connectivity and inter- and intra-hemispheric connections. The connectogram visualization was created using Circos (<http://circos.ca/>). Violin plots and permutation tests histograms of the significant between groups differences (p<0.05 FDR corrected) for the local network features of node strength **(B)** and clustering coefficients **(C)**. The histograms represent the t-value results of 1000 permutations tests, and the vertical red line represents the true t-value comparing the MDD vs. HC.

**Table S2. Regions with decreased connectivity with right CA3/4**

| ***ROI*** | ***t-value*** | ***p-value*** | ***Cohen’s d*** |
| --- | --- | --- | --- |
| Right Thalamus Proper | 2.80 | 0.004 | 0.62 |
| Right inferiorparietal | 1.92 | 0.03 | 0.33 |
| Right superiorparietal | 1.76 | 0.04 | 0.33 |
| Right subiculum | 1.84 | 0.04 | 0.38 |
| Right Lateral nucleus | 1.76 | 0.04 | 0.38 |
| Right Basal nucleus | 1.73 | 0.04 | 0.36 |

One sided two sample t-tests (MDD < HC) were conducted for right CA3/4 connectivity with all other ROIs in the network.
